# High-resolution vessel wall magnetic resonance imaging for the diagnosis of neurocysticercosis vasculitis

**DOI:** 10.1590/0037-8682-0203-2022

**Published:** 2022-09-26

**Authors:** Vinícius Ramos Daoud Yacoub, Marcelo de Carvalho Ramos, Fabiano Reis

**Affiliations:** 1 Universidade Estadual de Campinas, Faculdade de Ciências Médicas, Departamento de Anestesiologia, Oncologia e Radiologia, Campinas, SP, Brasil.; 2 Universidade Estadual de Campinas, Faculdade de Ciências Médicas, Departamento de Clínica Médica, Campinas, SP, Brasil.

A 48-year-old man presented with chronic headaches since 2014 in addition to dizziness, somnolence, and forgetfulness. He also reported episodes of transient focal neurological deficits, especially motor impairment, which resolved spontaneously.

An initial brain computed tomography (CT) scan revealed multiple parenchymal calcified nodules, a focal lesion with peripheral enhancement, and perilesional edema on the left frontal lobe. These findings, as well as the accompanying symptoms, were consistent with neurocysticercosis in the calcified nodular and colloidal vesicular stage.

**FIGURE 1:**
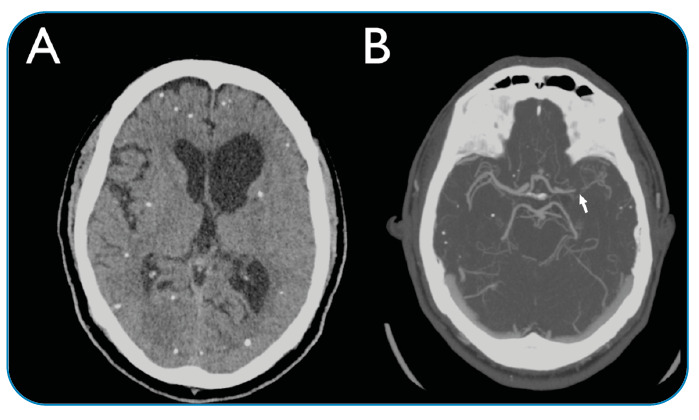
**(A)** Non-contrast-enhanced head computed tomography (CT) showing multiple sub-centimeter calcified lesions scattered throughout the supratentorial region of the brain. The presence of hydrocephalus is an important indicator of subarachnoid and intraventricular neurocysticercosis. **(B)** CT angiography of the cerebral arteries showing numerous focal stenoses in the left middle cerebral artery (arrow). Although not pathognomonic, this finding is highly indicative of arteritis and requires further investigation.

**FIGURE 2:**
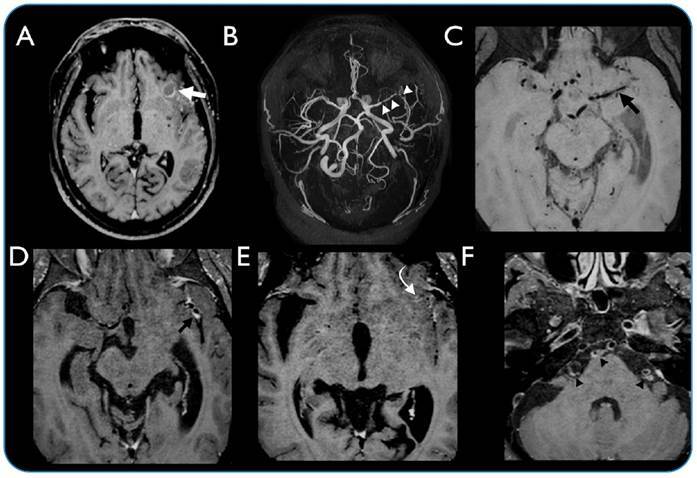
High-resolution vessel wall imaging (HR-VWI; Achieva 3.0 T; Philips Healthcare, The Netherlands; RT, 600 ms; ET, 29 ms; SPAIR; reconstruction matrix, 320; field of vision, 120 mm; voxel size 0.8 × 0.8 × 0.8 mm^3^; blood suppression, VISTA) in **(A)** (T1 axial post-contrast) showing a hypointense lesion with peripheral contrast enhancement, compatible with the vesicular colloidal stage of neurocysticercosis in the left frontal lobe (white arrow); **(B)** (maximum intensity projection arterial sequence SENSE) showing multifocal arterial narrowing in the left middle cerebral artery (white arrowheads); **(C)** (axial HR-VWI without contrast) and **(D)** (HR-VWI post-contrast) showing wall enhancement in the middle cerebral artery (black arrows); **(E)** (HR-VWI) showing leptomeningeal enhancement (curved arrow); **(F)** (HR-VWI post-contrast) showing enhancement in the wall of the basilar artery (black arrowheads).

Lumbar puncture investigations showed elevated cerebrospinal fluid opening pressure (30 cm H_2_O) and presence of white blood cells (51 cells/mm^3^; 78% lymphocytes). An angiographic CT scan revealed hydrocephalus and segmental narrowing of the left middle cerebral artery (Figure 1). High-resolution vessel wall imaging (HR-VWI) based on magnetic resonance imaging (MRI) indicated signs of arachnoiditis and findings consistent with vasculitis (Figure 2). The patient was treated with high-dose corticosteroid therapy and methotrexate (a steroid-sparing agent), and he is currently under hospital care.

The current diagnostic criteria for neurocysticercosis include clinical manifestations, previous exposure, and neuroimaging studies (CT and MRI)^1^. Subarachnoid neurocysticercosis is the most severe form of the disease and can lead to cerebrovascular complications involving multiple mechanisms, including arteritis, which may cause lacunar infarctions^2^. Conventional techniques for neurovascular imaging may fail to distinguish diseases within the vessel wall. HR-VWI is being increasingly used because the spatial resolution and enhancement patterns can depict vessel wall inflammation^3^. HR-VWI has high sensitivity in detecting arteritis and facilitates early diagnosis.
